# The high-pressure lithium–palladium and lithium–palladium–hydrogen systems

**DOI:** 10.1038/s41598-022-16694-2

**Published:** 2022-07-19

**Authors:** Mungo Frost, Emma E. McBride, Jesse S. Smith, Siegfried H. Glenzer

**Affiliations:** 1 High Energy Density Science Division, SLAC National Accelerator Laboratory, 2575 Sand Hill Road, Menlo Park, USA; 2 Stanford PULSE Institute, SLAC National Accelerator Laboratory, 2575 Sand Hill Road, Menlo Park, USA; 3 High Pressure Collaborative Access Team, X-ray Science Division, Argonne National Laboratory, Argonne, USA

**Keywords:** Structure of solids and liquids, Structure of solids and liquids

## Abstract

The lithium–palladium and lithium–palladium–hydrogen systems are investigated at high pressures at and above room temperature. Two novel lithium–palladium compounds are found below ${18.7}\,{\mathrm{GPa}}$. An ambient temperature phase is tentatively assigned as $F{\bar{4}}3m\,\hbox {Li}_{17}\hbox {Pd}_{4}$, with $a = 17.661(1)$ Å at 8.64 GPa, isostructural with $\hbox {Li}_{17}\hbox {Sn}_{4}$. The other phase occurs at high-temperature and is $I{\bar{4}}3m\, \hbox {Li}_{11}\hbox {Pd}_{2}$, $a = 9.218(1)$ Å at 3.88 GPa and 200 $^\circ {\mathrm{C}}$, similar to $\hbox {Li}_{11}\hbox {Pt}_{2}$, which is also known at high pressure. The presence of hydrogen in the system results in an $I{\bar{4}}3m$ structure with $a = 8.856(1)$ Å at 9.74 GPa. This persists up to ${13.3}\,\mathrm{GPa}$, the highest pressure studied. Below ${2}\,{\mathrm{GPa}}$ an *fcc* phase with a large unit cell, $a = 19.324(1)$ Å at 0.39 GPa, is also observed in the presence of hydrogen. On heating the hydrogen containing system at 4 GPa the $I{\bar{4}}3m$ phases persists to the melting point of lithium. In both systems melting the lithium results in the loss of crystalline diffraction from palladium containing phases. This is attributed to dissolution of the palladium in the molten lithium, and on cooling the palladium remains dispersed.

## Introduction

Lithium and hydrogen are low Z elements which exhibit complex behaviors at high density, in part due to quantum mechanical effects arising from their low masses^1,2^. Both have maxima in their melting curves^3–5^ and exhibit complex low symmetry crystal structures, despite their apparent simplicity at low pressure^6,7^. High pressure metal hydrides have attracted considerable attention recently as various novel compounds have been found to form only at high-pressure^8,9^, some of which have record breaking high Tc superconductivity^10,11^.

The alkali metals have also been found to form novel intermetallics at high pressure^12–16^, with a number of high pressure lithides recently reported^14–16^. Alkali metals are more electropositive than many other metals, particularly the noble metals, and charge transfer from the alkali metal to the other component of the intermetallic plays a role in their stability^14,17^.

Various transition metal – lithium intermetallics are reported in the literature at ambient pressure. Typically, these are synthesized by heating the reactants considerably above the melting point of lithium (180 $^\circ {\mathrm{C}}$ at ambient pressure), under an inert atmosphere with characterization performed on quenched products. These have attracted attention due to their potential application in energy storage materials as anode materials for lithium ion batteries^18^. Compared to current intercalation type compounds they offer potentially much higher lithium content and hence energy storage potential^15,18^, motivating the exploration of lithium-rich intermetallic compounds.

Palladium is a transition metal widely used in catalysis and hydrogen purification. The hydrogen affinity of palladium is very sensitive to pressure with palladium hydride, $\hbox {PdH}_x$, forming below 1 bar. The hydrogen content increases from $x=0.6$ at formation to $x=1$ at a few GPa^19^. Further compression has not been observed to result in any higher hydride to ${100}\,{\mathrm{GPa}}$^20^. A number of lithium palladium intermetallics have been reported at ambient pressure with stoichiometries of $\hbox {LiPd}_{7}$, $\hbox {LiPd}_{2}$, $\hbox {LiPd}$, $\hbox {Li}_{2}\hbox {Pd}$, $\hbox {Li}_{3}\hbox {Pd}$, and $\hbox {Li}_{15}\hbox {Pd}_{4}$^21^. All of these were formed at high temperature and quenched to ambient.

Palladium lithium hydride, $\hbox {PdLiH}_x$, $0.7< x < 1$, is also known and has been calculated to exhibit superconductivity with the low mass lithium making a large contribution to the electron-phonon coupling^22^. Subsequent studies have synthesized it but not observed superconductivity. Various synthesis conditions have been used including sintering equimolar mixtures of LiH and Pd in a hydrogen atmosphere at 10 bar^23^, heating LiPd in ${270}\,{\mathrm{MPa}}$ of hydrogen gas^24^, and compressing a sealed mixture of LiH and Pd to ${3}\,{\mathrm{GPa}}$ and heating to ${773}\,{\mathrm{K}}$^25^. These all yield *P*4/*mmm* $\hbox {LiPdH}_x$ with *x* close to 1. Cooling to ${4}\,{\mathrm{K}}$ at ambient pressure^23^, or at ${270}\,{\mathrm{MPa}}$^24^ did not result in superconductivity. Liu et al.^25^ measured resistivity as a function of temperature and pressure from 2 to ${300}\,{\mathrm{K}}$ up to ${25.2}\,{\mathrm{GPa}}$ and observe a minimum in the resistivity with pressure at ${18.3}\,{\mathrm{GPa}}$ but no superconductivity. They speculate that the discrepancy between theory and experiment may arise from scattering by impurities or hydrogen vacancies.

The application of pressure allows the exploration of exotic chemistry and materials synthesis which does not occur under ambient conditions. To date no study has considered the effect of pressure on the lithium–palladium system, or the lithium–rich lithium–palladium–hydrogen system, nor reported ambient temperature reaction of Pd and Li. Platinum, which lies directly below palladium in the periodic table and shares many properties with it, has been studied under pressure with lithium. It was found to form a $\hbox {Li}_{11}\hbox {Pt}_{2}$ compound with space group $I{\bar{4}}3m$ below ${11}\,{\mathrm{GPa}}$, above which it expels lithium to form *P*6/*mmm* $\hbox {Li}_{2}\hbox {Pt}$^14^.

Here we study the lithium–rich palladium–lithium and palladium–lithium–hydrogen systems at high pressure, at and above room temperature. All samples exhibited reactions in the cells as loaded, prior to further compression. The initial pressures varied from 0.4 to ${6}\,{\mathrm{GPa}}$. We observe two novel compounds which are isostructural with lithium compounds known in other systems. The presence of hydrogen changes the structure adopted by the compound showing it to play a role in the properties of the system. This also demonstrates that the hydrogen affinity of the compound formed is higher than that of pure lithium, which was present in excess in all samples.

## Results

### Lithium–palladium

**Figure 1:**
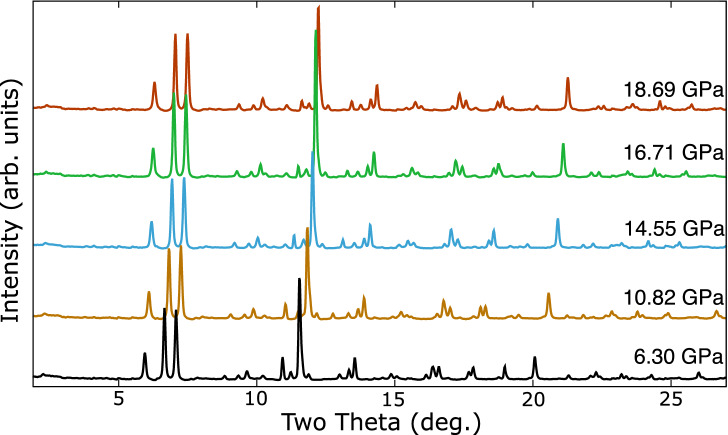
Pressure evolution of the background subtracted integrated diffraction pattern of the lithium–palladium intermetallic at ambient temperature.

In the absence of hydrogen the lithium and palladium had already reacted at after a few days at ${5.0}\,{\mathrm{GPa}}$, the lowest pressure measured. Figure 1 shows the pressure evolution of the integrated diffraction patterns collected with 0.4246 Å radiation. These indicate no phase transitions below ${18.7}\,{\mathrm{GPa}}$ at ambient temperature. The observed peaks cannot be fitted to any known palladium–lithium or platinum–lithium intermetallic. A weak peak at $2\theta =2.26^\circ$, corresponding to $d = 10.77$ Å, at ${6.30}\,{\mathrm{GPa}}$ indicates a large unit cell.

**Figure 2:**
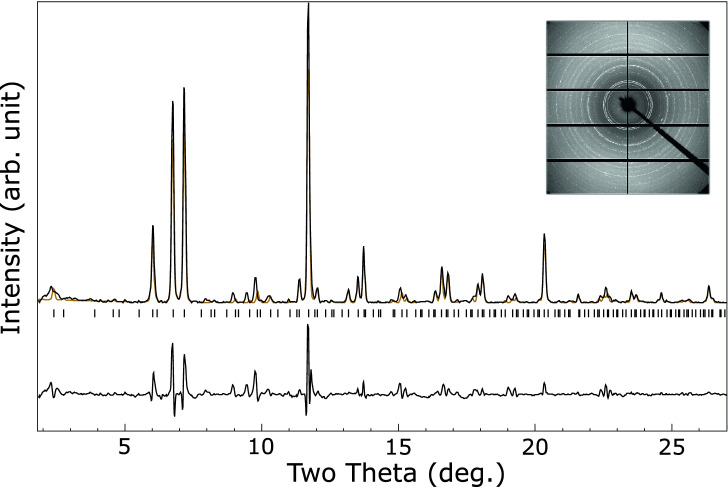
Integrated diffraction pattern of the lithium–palladium intermetallic at ambient temperature and ${8.64}\,{\mathrm{GPa}}$ (black), with LeBail fit to $F{\bar{4}}3m$ phase with $a = 17.661$ Å  (orange) and residual on same scale (lower black line). Allowed peak positions are indicated by ticks. See text for discussion of fit. *Inset:* Unintegrated diffraction pattern.

**Figure 3:**
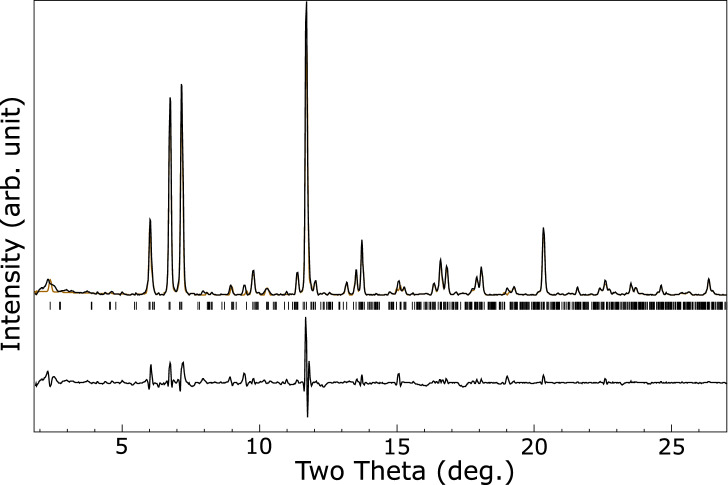
Integrated diffraction pattern of the lithium–palladium intermetallic at ambient temperature and ${8.64}\,{\mathrm{GPa}}$ (black), with LeBail fit to a face centered tetragonal phase with $a = 17.656$ and $c = 17.868$ Å  (orange) and residual on same scale (lower black line). Allowed peak positions are indicated by ticks. The unit cell is expressed as face centered tetragonal rather than the equivalent, smaller volume, body centered tetragonal cell to aid comparison to the $F{\bar{4}}3m$ phase fitted in Fig. 2.

Figure 2 shows a LeBail fit^26^ to an $F{\bar{4}}3m$ structure with $a = 17.661(1)$ Åat ${8.64}\,{\mathrm{GPa}}$. This is similar to known $\hbox {Li}_{17}\hbox {M}_{4}$ structures with M = Ge, Sn, Pb, which have $F{\bar{4}}3m$ symmetry and ambient pressure lattice parameters of 18.756, 19.690, and 19.842 Å respectively^27^. Close inspection of the LeBail fit reveals a few peaks which are not well fitted, particularly in the region of 8.5 to 10.5 degrees. The lithium–palladium system is known to form numerous intermetallics at ambient pressure and some of these peaks may be due to other low-concentration phases. We do note that a slight tetragonal distortion of the unit cell with $a = 17.656$ Å and $c = 17.869$ Å results in a much improved fit, see Fig. 3, but the symmetry lowering also results in many allowed reflections so this could be pathological. A similarly improved fit, but with even more possible peaks, may be obtained by a monoclinic distortion. The $F{\bar{4}}3m$ $\hbox {Li}_{17}\hbox {M}_{4}$ type structure is the best fit we can obtain to the high-pressure lithium palladium compound, and we tentatively suggest that the structure is this, or closely related to it.

**Figure 4:**
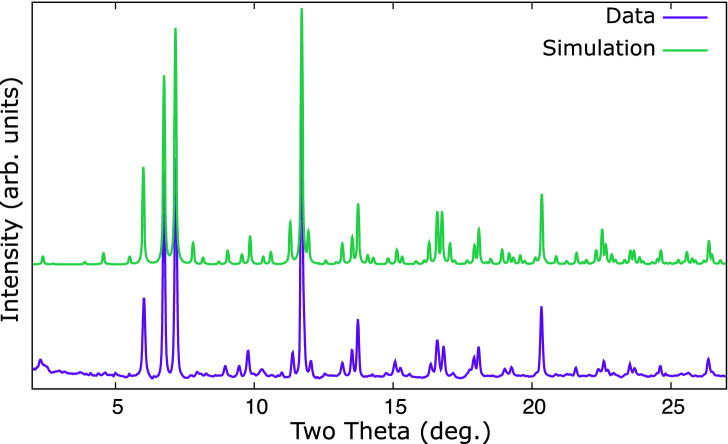
Comparison of observed data from a lithium–palladium mixture at ${8.64}\,{\mathrm{GPa}}$ (lower trace) and a simulated powder pattern (upper trace) of $F{\bar{4}}3m$ $\hbox {Li}_{17}\hbox {Pd}_{4}$ using the atomic parameters from $\hbox {Li}_{17}\hbox {Sn}_{4}$^27^ and lattice parameter and radiation from this study. The similarity supports the novel palladium–lithium compound having a similar structure to $\hbox {Li}_{17}\hbox {Sn}_{4}$.

The $\hbox {Li}_{17}\hbox {M}_{4}$ structure has $Z = 20$ for a total of 420 atoms per unit cell. This extremely complex structure was previously determined via single crystal X-ray diffraction^27^. Powder X-ray diffraction in DACs suffers from limited angular range imposed by the geometry of the cell, and limited resolution causing higher angle peaks to overlap. Along with the potential for contaminant peaks this makes a full Rietveld refinement impractical, however a powder X-ray diffraction pattern of the proposed structure can be simulated. Figure 4 compares the observed data with a simulated pattern produced using the literature atomic parameters for $\hbox {Li}_{17}\hbox {Sn}_{4}$^27^, but with Sn exchanged for Pd and the lattice parameter set to match that LeBail fitted to the observed data. The patterns are quite similar which supports the $F{\bar{4}}3m$ $\hbox {Li}_{17}\hbox {Pd}_{4}$ structural candidate for the novel palladium lithide.

**Figure 5:**
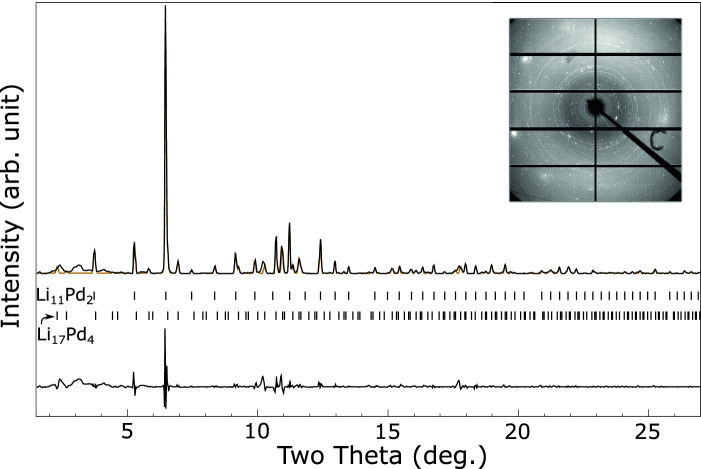
Integrated diffraction pattern of the lithium–palladium intermetallics at 200 $^\circ$C and ${3.88}\,{\mathrm{GPa}}$ (black), with LeBail fit to proposed ambient-temperature $F{\bar{4}}3m$ $\hbox {Li}_{17}\hbox {Pd}_{4}$ ($a = 18.229$ Å) and high-temperature $I{\bar{4}}3m$ $\hbox {Li}_{11}\hbox {Pd}_{2}$ ($a = 9.218$ Å) phases (orange) and residual on same scale (lower black line). Allowed peak positions for each phase are indicated by ticks. The broad unfitted features at low angle are from the Kapton window of the heater. *Inset:* Unintegrated diffraction pattern.

On heating at ${4}\,{\mathrm{GPa}}$ the appearance of the pattern changes with new peaks appearing at 200 $^\circ$C, see Fig. 5. This pattern can be fitted as a mixture of the ambient temperature $F{\bar{4}}3m$ phase and a *bcc* phase with $a = 9.218(1)$ Å at ${3.88}\,{\mathrm{GPa}}$. This is compatible with $I{\bar{4}}3m$ symmetry and consideration of the density suggests it is $\hbox {Li}_{11}\hbox {Pd}_{2}$, isostructural with $\hbox {Li}_{11}\hbox {Pt}_{2}$ observed in the high pressure lithium–platinum system^14^. This phase has a $\gamma$-brass structure and has been observed in other palladium-group intermetallics^28,29^.

Further heating to 225 $^\circ$C melts the lithium and leads to the loss of all crystalline diffraction from palladium. We attribute this to dissolution of the palladium into the large excess of lithium. On cooling, peaks from *bcc*-lithium reappear, however, those from the palladium intermetallic do not, nor do any new peaks appear. The most likely explanation for the loss of diffraction from the palladium is that it is incorporated into the large excess of *bcc* lithium. Further compression causes the *bcc* to *fcc* transition in lithium near ${7.5}\,{\mathrm{GPa}}$, as expected^30^, but no peaks other than those of *fcc*-lithium emerge implying that the palladium remains dispersed.

### Lithium–palladium–hydrogen

**Figure 6:**
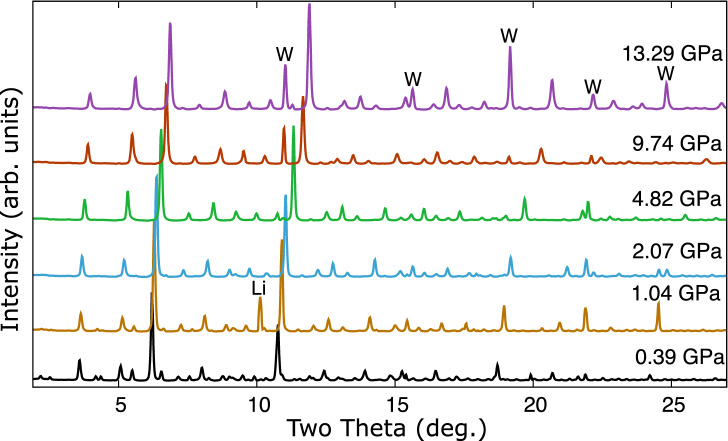
Pressure evolution of the background subtracted integrated diffraction patterns of the lithium–palladium–hydrogen system at ambient temperature. The peaks marked W in the highest trace are due to tungsten and are present at similar angles in some lower pressure traces. A strong lithium peak is also marked at ${1.04}\,{\mathrm{GPa}}$.

**Figure 7:**
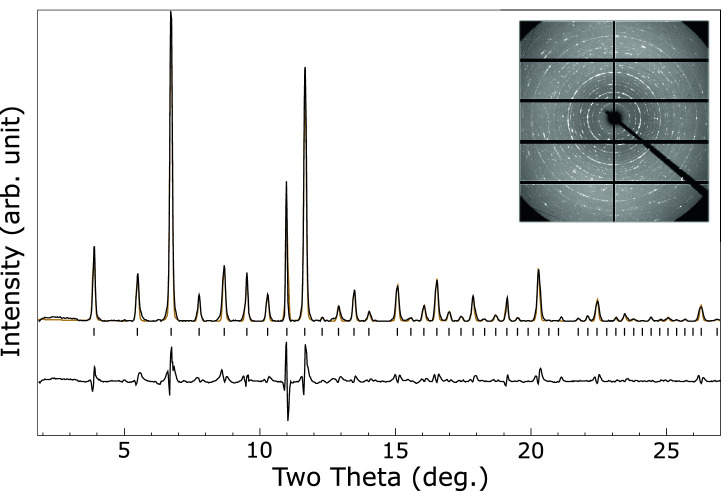
Integrated diffraction pattern of the lithium–palladium–hydrogen intermetallic at ambient temperature and ${9.74}\,{\mathrm{GPa}}$ (black), with LeBail fit to $I{\bar{4}}3m$ phase ($a = 8.856$ Å) (orange) and residual on same scale (lower black line). Allowed peak positions are indicated by ticks. *Inset:* Unintegrated diffraction pattern.

**Figure 8:**
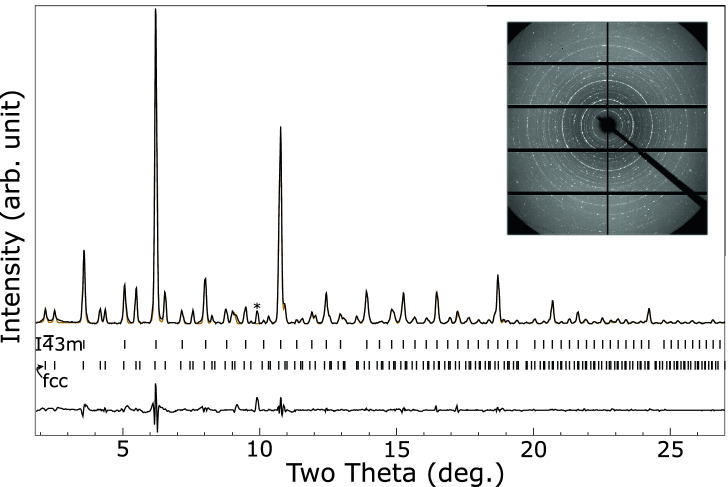
Integrated diffraction pattern of the lithium–palladium–hydrogen intermetallics at ambient temperature and ${0.39}\,{\mathrm{GPa}}$ (black), with LeBail fit to the low-pressure *fcc* phase ($a = 19.324$ Å) and the high-pressure $I{\bar{4}}3m$ phase ($a = 9.597$ Å) (orange) and residual on same scale (lower black line). Allowed peak positions for each phase are indicated by ticks. The unfitted peak marked with an asterisk is the $\langle 110\rangle$ reflection from *bcc*-lithium. *Inset:* Unintegrated diffraction pattern.

**Figure 9:**
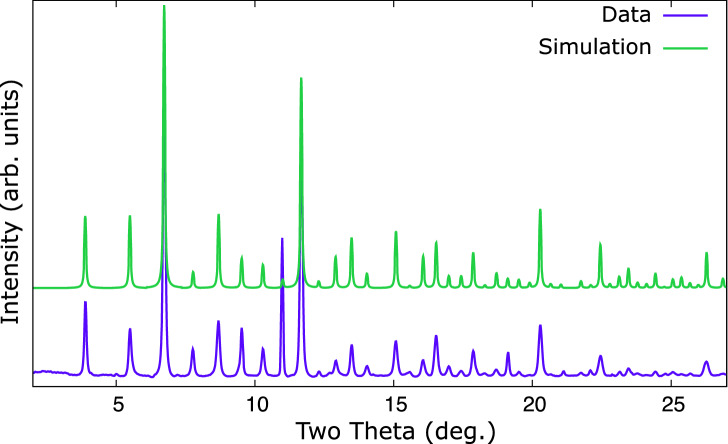
Comparison of observed data from a lithium–palladium–hydrogen mixture at ${9.74}\,{\mathrm{GPa}}$ (lower trace) and a simulated powder pattern (upper trace) of $I{\bar{4}}3m$ $\hbox {Li}_{11}\hbox {Pd}_{2}$ using the atomic parameters from $\hbox {Li}_{11}\hbox {Pt}_{2}$^14^ and lattice parameter and radiation from this study. The similarity supports the palladium–lithium–hydrogen compound having a similar structure to $\hbox {Li}_{11}\hbox {Pt}_{2}$.

The presence of hydrogen in cells loaded with palladium, lithium and lithium hydride stabilized different phases under pressure, with a transition occuring between 1 and ${2}\,{\mathrm{GPa}}$, see Fig. 6. Above the transition a single phase is observed. This is well fitted by a *bcc* structure with and $a = 8.856(1)$ Å at ${9.74}\,{\mathrm{GPa}}$, compatible with $I{\bar{4}}3m$ symmetry, similar to the $\hbox {Li}_{11}\hbox {Pd}_{2}$ phase observed at high-temperature in the lithium–palladium system. An integrated pattern and LeBail fit are shown in Fig. 7. Below ${2}\,{\mathrm{GPa}}$ a second set of peaks are observed, see Fig. 6, which are fitted by an *fcc* phase with a large unit cell of $a = 19.324(1)$ Å at ${0.39}\,{\mathrm{GPa}}$. A LeBail fit is shown in Fig. 8. The stoichiometry of the compounds cannot be determined, though hydrogen must be present to cause the structural differences from the lithium–palladium system. Figure 9 shows the similarity between observed diffraction pattern of the *bcc* phase and simulated powder data based on the structure of $I{\bar{4}}3m$ $\hbox {Li}_{11}\hbox {Pt}_{2}$^14^.

Between ${0.4}\,{\mathrm{GPa}}$, the lowest pressure the palladium, lithium and lithium hydride system was studied at, and ${1}\,{\mathrm{GPa}}$ an additional *fcc* phase with a large unit cell is present, see fit in Fig. 8. This is similar to the $F{\bar{4}}3m$ $\hbox {Li}_{17}\hbox {Pd}_{4}$ phase proposed for the lithium–palladium system, and may be a related structure. The peaks from this phase are weak by ${1}\,{\mathrm{GPa}}$ and absent at ${2}\,{\mathrm{GPa}}$.

On heating at ${4}\,{\mathrm{GPa}}$, the hydride containing sample showed no phase changes below the melting point of lithium. On melting the lithium, the palladium dissolves, as for the lithium–palladium system, and remains dispersed on cooling.

## Discussion

Without hydrogen the lithium–palladium system forms an ambient temperature structure we tentatively assign to be of $F{\bar{4}}3m$ symmetry and isostructural with $\hbox {Li}_{17}\hbox {Sn}_{4}$. Interpretation of the powder pattern, Fig. 2, is hindered by the possibility of additional phases or small distortions of the unit cell. Comparison to a simulated pattern for $\hbox {Li}_{17}\hbox {Pd}_{4}$, Fig. 4, suggests the observed structure is the same, or closely related. This structure has not been observed in any other transition metal - alkali metal intermetallic, however it is observed in the lithides of silicon, germanium, tin and lead^27,31^ with slightly varying stoichiometry.

**Figure 10:**
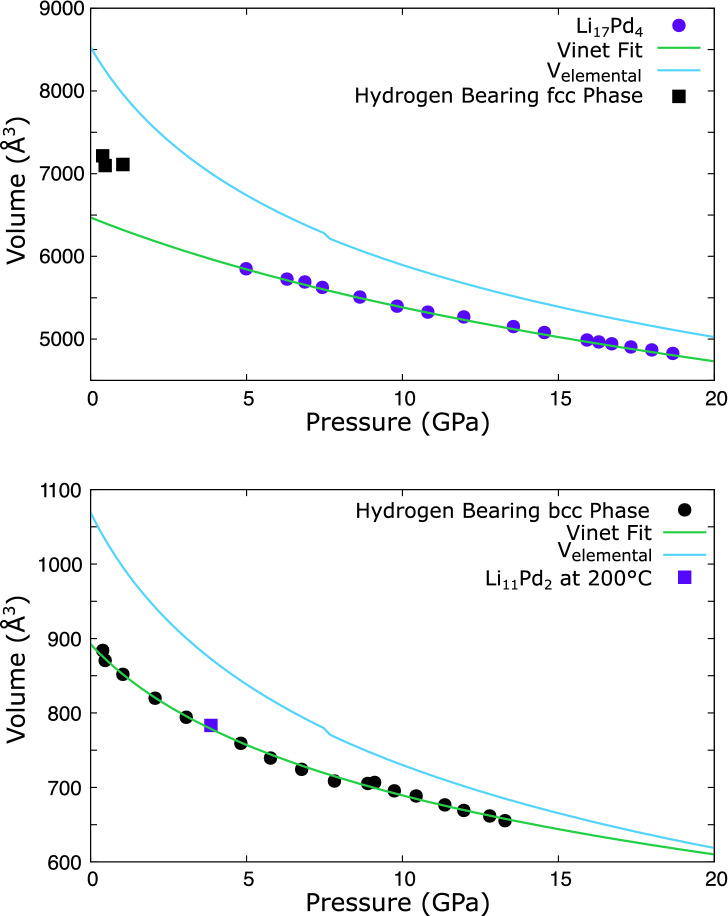
Volume vs pressure plots. Top: the $\hbox {Li}_{17}\hbox {Pd}_{4}$ $F{\bar{4}}3m$ phase (fuchsia circles) and low-pressure *fcc* hydrogen bearing phase (black squares). The green line is a Vinet fit to the circle points, note that hydrogen bearing *fcc* phase lies off this curve. The blue line is the volume of an equivalent number of lithium (340) and palladium (80) atoms in the elemental form^20,30,32^. Bottom: the *bcc* hydrogen bearing phase (black circles) and $\hbox {Li}_{11}\hbox {Pd}_{2}$ $I{\bar{4}}3m$ phase at 200 $^\circ \mathrm{C}$ (fuchsia square). The green Vinet fit is to the circle points. The blue line is the volume of 44 lithium and 8 palladium atoms in elemental form, corresponding to $\hbox {Li}_{11}\hbox {Pd}_{2}$. The discontinuity near ${8}\,{\mathrm{GPa}}$ lies close to the *bcc* to *fcc* transition in lithium and may indicate a change in stoichiometry.

Volumetric considerations give further support for the structures of both $\hbox {Li}_{17}\hbox {Pd}_{4}$ and $\hbox {Li}_{11}\hbox {Pd}_{2}$ assigned from comparison of the diffraction data to other known lithides. Figure 10 shows pressure-volume data collected on compression for both phases, complete with fitted equations of state of the Vinet form:

$$\begin{aligned} P = 3 B_0 \left( \frac{1-\left( \frac{V}{V_0}\right) ^{\frac{1}{3}}}{\left( \frac{V}{V_0}\right) ^{\frac{2}{3}}} \right) e^{\frac{3}{2} \left( B_0' - 1\right) \left( 1-\left( \frac{V}{V_0}\right) ^{\frac{1}{3}}\right) } \end{aligned}$$

Where $B_0$ and $V_0$ are the zero-pressure bulk modulus and volume, *V* is the volume at pressure *P*, and $B_0'$ is the pressure derivative of the bulk modulus. The fitted equation of state parameters are given in Table 1. Both compounds exhibit intermediate compressibility between those of lithium, which is highly compressible^30,32^, and palladium, which has low compressibility^20^.

**Table 1:** Fitted Vinet equation of state parameters.

Phase	${V_0}$(Å${^3}$)	${B_0}$ (GPa)	${B_0'}$
$F{\bar{4}}3m$ $\hbox {Li}_{17}\hbox {Pd}_{4}$	6471(48)	43.9(39)	2.63(42)
*bcc* hydrogen bearing phase	892.6(92)	19.6(29)	5.45(72)

Figure 10 also shows the pressure-volume curves for the pure elements at the proposed stoichiometries. In both cases the compounds are denser, with the difference decreasing at higher pressure. Table 2 gives the ratio of the volume of palladium–lithium intermetallics to their elemental constituents and shows the increased density of the intermetallics in this study to be within the range of expected values. In the solid phase the pressure-volume curve of a compound is expected to lie close to that of its constituents of enthalpic grounds. Excess volume will have an increasingly punitive *PV* term in its free energy, while overly reduced volume would lead to unphysical densities.

**Table 2:** Comparison of the ratio of the volume of various lithium–palladium intermetallics to the volume of an equivalent quantity of elemental lithium and palladium.

Compound	${V/V_{elemental}}$	Conditions	Ref.
$F{\bar{4}}3m$ $\hbox {Li}_{17}\hbox {Pd}_{4}$	0.76	0 GPa	This study
$F{\bar{4}}3m$ $\hbox {Li}_{17}\hbox {Pd}_{4}$	0.87	5 GPa	This study
$F{\bar{4}}3m$ $\hbox {Li}_{17}\hbox {Pd}_{4}$	0.91	10 GPa	This study
$I{\bar{4}}3m$ $\hbox {Li}_{11}\hbox {Pd}_{2}$	0.90	3.86 GPa, 200 $^\circ$C	This study
$I{\bar{4}}3d$ $\hbox {Li}_{15}\hbox {Pd}_{4}$	0.71	Ambient	^21^
$Fm{\bar{3}}m$ $\hbox {Li}_{3}\hbox {Pd}$	0.74	Ambient	^21^
*P*6/*mmm* $\hbox {Li}_{2}\hbox {Pd}$	0.73	Ambient	^21^
$Pm{\bar{3}}m$ LiPd	0.73	Ambient	^21^
$P{\bar{6}}$ LiPd	0.75	Ambient	^21^
$Fm{\bar{3}}m$ $\hbox {LiPd}_{7}$	0.90	Ambient	^21^

Temperature is ambient except where noted.

Volumes for $\hbox {Li}_{17}\hbox {Pd}_{4}$ from this study are based on its equation of state.

In the presence of hydrogen two phases are observed. At low pressure an *fcc* phase with a large unit cell ($a = 19.324$ Å at ${0.39}\,{\mathrm{GPa}}$) coexists with a phase which appears similar to the $I{\bar{4}}3m$ $\hbox {Li}_{11}\hbox {M}_{2}$ phase observed in the lithium–platinum system^14^ and lithium–palladium at high-pressure and high-temperature. Above 1 GPa the *fcc* phase is lost and only the $I{\bar{4}}3m$ phase remains. The volume of the low pressure *fcc* phase observed in the hydride containing system lies off the curve of the $\hbox {Li}_{17}\hbox {Pd}_{4}$ produced without hydrogen present and both cannot be fitted to a single equation of state. This could arise from hydrogen absorbed into the lattice, or may indicate they have different structures.

Both lithium and hydrogen are low *Z* and so scatter X-rays very weakly compared to palladium. Therefore, we would not expect to be able to resolve partial replacement of lithium by hydrogen, or additional hydrogen incorporated into the lattice, except on volumetric grounds. This makes the stoichiometries of the phases formed in the palladum–lithium–hydrogen samples impossible to determine, and non-stoichiometric phases cannot be ruled out. Examination of the patterns and comparison to the high temperature lithium–palladium system, and to the literature on the lithium–platinum system, strongly supports the structure being an analog to the $I{\bar{4}}3m$ $\hbox {Li}_{11}\hbox {M}_{2}$ (M = Pt, Pd) $\gamma$-brass structure. The high-temperature $I{\bar{4}}3m$ $\hbox {Li}_{11}\hbox {Pd}_{2}$ phase which forms in the absence of hydrogen has similar volume to the hydrogen bearing $I{\bar{4}}3m$ phase, see Fig. 10. This limits the quantity of additional hydrogen which can be present and suggests that the structural differences are caused either by substitution of lithium with hydrogen, or very dilute hydrogen present in the lattice. The inclusion of trace impurities has previously been suggested to change the structure of LiPd at ambient pressure^21^. Inspection of the pressure-volume data of the hydrogen containing $I{\bar{4}}3m$ structure shows a slight discontinuity at ${8}\,{\mathrm{GPa}}$, this is likely due to small compositional changes such as the incorporation of additional hydrogen, as the diffraction patterns above and below this appear similar.

A $\hbox {Li}_{15}\hbox {Pd}_{4}$ palladium–lithium intermetallic with $I{\bar{4}}3d$ symmetry has been reported at ambient pressure^21^ with $a = 10.676$ Å. This is not compatible with any diffraction pattens observed in this study. The high-temperature $\hbox {Li}_{11}\hbox {Pd}_{2}$ and related hydrogen containing $I{\bar{4}}3m$ phase both have several peaks which are incompatible with the $I{\bar{4}}3d$ space group. Van Vucht and Buschow^21^ observed an unknown F-centered cubic lithium–palladium intermetallic with a = 19.009 Å at 10 to 16 at% Pd. While they did not observe satisfactory agreement with the $\hbox {Li}_{17}\hbox {Pb}_{4}$ structure (wrongly assigned as the $\hbox {Li}_{22}\hbox {Pb}_{5}$ structure at the time of their publication^27^) it is possible their *fcc* phase is the same as, or related to, the one we observe here at higher pressure.

The study of transition metal hydrides and lithides is of interest to various fields including hydrogen storage^33^, battery technology^18,34^ and superconductivity^22^. The phases reported here present a test for theory at high pressure, particularly given the large body of work which exists on the binary hydrides of lithium and palladium^19,20,33,35^. The reactivity of lithium, lithium hydride and palladium is also relevant to hydrogen storage as both lithium and palladium have proposed applications in the hydrogen economy^33,36^.

In conclusion, we investigated the palladium–lithium and palladium–lithium–hydrogen systems to 18.7 and ${13.3}\,{\mathrm{GPa}}$ respectively. The palladium–lithium system reveals a single intermetallic at ambient temperature which we tentatively assign to an $F{\bar{4}}3m$ $\hbox {Li}_{17}\hbox {M}_{4}$ type structure, possibly with a small distortion. At 200 $^\circ$C at ${3.87}\,{\mathrm{GPa}}$ this partially converts to an $I{\bar{4}}3m$ $\hbox {Li}_{11}\hbox {Pd}_{2}$ structure analogous to $\hbox {Li}_{11}\hbox {Pt}_{2}$ in the dense lithium–platinum system. The palladium–lithium–hydrogen system forms an $I{\bar{4}}3m$ structure similar to $\hbox {Li}_{11}\hbox {Pd}_{2}$ at all pressures and temperatures studied. Below ${2}\,{\mathrm{GPa}}$ we also observe an *fcc* phase with a large unit cell in this system. Due to the weak scattering from hydrogen and lithium it is not possible to determine the stoichiometries, though volumetric considerations disallow high concentrations of additional hydrogen in the $I{\bar{4}}3m$ phase. On heating to the melting point of lithium, diffraction from the palladium compounds disappears in both systems and does not reappear on cooling. This is attributed to the palladium forming a dilute solid solution in the large excess of lithium metal.

## Methods

Diamond anvil cells (DACs) were prepared with rhenium gaskets on Boehler-Almax type diamond anvils with culet sizes ranging from 250 to ${400}\,{\upmu {\mathrm{m}}}$. Pressure was determined to an uncertainty of ${0.1}\,{\mathrm{GPa}}$ via the temperature adjusted equation of state^37^ of a small quantity of tungsten powder (Alfa Aesar, 99.9%) added to each loading. Tungsten was chosen as it is known not to react with lithium^4,30^, and does not form hydrides below 25 GPa^9^. Powdered palladium (Alfa Aesar, 99.95%) was added to each cell such that the lithium would be in large excess. Lithium metal (Alfa Aesar, 99.9%) and, for hydrogen containing cells lithium hydride (Alfa Aesar, 99.4%), were loaded under a high purity argon atmosphere. Hydrogen was introduced as lithium hydride because lithium metal fully reacts with hydrogen gas at pressures below ${50}\,{\mathrm{MPa}}$^35^ making gas loading an unnecessary complication. The maximum pressure was ${18.7}\,{\mathrm{GPa}}$ to avoid damage to the diamond anvils by lithium at higher pressure^3,30,32^. There was no evidence of reactions involving the tungsten pressure marker, diamond anvils or rhenium gasket in any cell, nor was there any observable reaction between the lithium and lithium hydride.

Angular dispersive powder X-ray diffraction measurements were carried out at APS beamline 16-ID-B (HPCAT) using 0.4246 Å radiation. Full diffraction maps were taken by rastering the samples with ${5}\,{\upmu }{\mathrm{m}}$ steps taking a diffraction pattern at each location to ensure purity of the loadings. The load on the cells was controlled using a gas membrane with patterns collected on a Dectris Pilatus 1M large area detector with a sample to detector distance of ${200}\,{\mathrm{mm}}$ and ${172}\,{\upmu }{\mathrm{m}}$ pixels. Resistive heating was performed under vacuum with Kapton X-ray windows using the HPCAT resistive heating setup^38^. Temperature was determined with uncertainty of ± 3 $^\circ$C using two type K thermocouples in contact with the cell. Ambient temperature runs were performed in air without the Kapton windows.

Diffraction patterns were integrated using the dioptas software package^39^. The integrated patterns were further analyzed using jana06^40^ and powdercell^41^. LeBail fits determine the unit cell parameters based on the angles of the powder diffraction peaks and can give information on the space group based on systematic absences^26^, but do not offer details on the location of atoms within the cell. LeBail fits were performed as the complex structures of the intermetallics formed, and limited q-range and resolution possible in diamond anvil cells, made Rietveld fits unstable. Strain and preferred orientation also cannot be ruled out, particularly as the compounds were formed in situ under pressure.

## Data Availability

The datasets used and/or analysed during the current study available from the corresponding author on reasonable request.
